# From coordination problems to complexity processing – second-order observation of inter-administrative relations

**DOI:** 10.3389/fsoc.2026.1872046

**Published:** 2026-06-19

**Authors:** Paul R. Schreiber, Aladin El-Mafaalani

**Affiliations:** Fakultät Sozialwissenschaften, Technische Universität Dortmund, Dortmund, Germany

**Keywords:** actor-centered institutionalism, complexity, decision-making, inter-administrative relations, public administration, second-order observation, social systems theory

## Abstract

This paper offers a conceptual analysis of inter-administrative relations by reconstructing them as an object constituted through analytical observation. Drawing explicitly on Niklas Luhmann’s Social Systems Theory, it employs second-order observation as a methodological framework to examine how inter-administrative relations are made observable within different analytical traditions. It first analyzes how inter-administrative relations are constituted within Actor-Centered Institutionalism, showing that they emerge through the distinction between coordination success and coordination failure and appear predominantly as problems or solutions of policy implementation. In a second step, systems theory is employed as a reflexive observational methodology. Using the system/environment distinction, public administration is observed as a decision system. Inter-administrative relations can then be described as forms that stabilize connectivity between differentiated decision contexts under conditions of environmental complexity. By systematically varying the observational distinctions through which inter-administrative relations are constituted, the paper demonstrates that they are not given empirical objects but take on different analytical forms depending on how they are observed: either as a normative category for evaluating coordination or as conditions of organizational decision-making. In this way, the contribution advances both systems-theoretical and administrative research. It demonstrates how second-order observation can be used as a transdisciplinary methodological tool to reconstruct and compare established analytical frameworks. It further shows that empirical diagnoses and reform proposals are effects of specific observational distinctions.

## 1 Inter-administrative relations as an object of second-order observation

Public administration in contemporary societies operates under conditions of extensive interdependence (Scharpf et al., 1976; Scharpf, 1978, 1985; Benz, 1997). Tasks are distributed across multiple organizations and levels of government, such that policy implementation depends on the alignment of decisions across administrative boundaries (Marks et al., 1996; Hooghe and Marks, 2001). These interdependencies are conceptualized within a specific strand of administrative research as *inter-administrative relations* (IARs), referring to observable connections between administrative actors involved in policy implementation (Bogumil and Kuhlmann, 2022; Bogumil and Gräfe, 2023; Oehlert and Kuhlmann, 2024). *Actor-centered institutionalism* (ACI) provides a widely used framework for making visible and analyzing these interdependencies between administrative actors in policy implementation (Mayntz and Scharpf, 1995; Scharpf, 1997). Within this framework, IARs are constituted as configurations of actors whose actions must be coordinated under institutional constraints. This raises the question of how IARs are constituted as an object of observation within such analytical frameworks. Since, as Baecker (1999) puts it, “one cannot proceed to construct anything, indeed to do anything, without drawing a distinction” (1999, 2), the distinction itself must become an object of observation.

This paper employs social systems theory (SST) in a reflexive capacity (Luhmann, 2002b, 96). It serves as a second-order observation of the ACI perspective on IARs (2.) and, in a further step, introduces an alternative observational distinction through which IARs are reconstructed (3.). While first-order observation describes the world by means of a form, understood in a Luhmannian sense as a distinction that renders phenomena observable through a specific selection (Luhmann, 2012, 113–119), second-order observation examines the distinctions employed by observers themselves and thereby makes observable the contingency and blind spots of these forms (Luhmann, 2002b, 86). Drawing on second-order observation, the reconstruction focuses on the distinctions through which IARs are constituted. In this sense, the ACI framework itself can already be understood as a form of second-order observation insofar as it employs distinctions through which administrative processes become observable while simultaneously producing characteristic blind spots.^1^

The paper therefore relates two different ways of rendering IARs observable. In both cases, IARs are constituted through guiding distinctions that become accessible through second-order observation. However, these distinctions differ in their analytical and normative orientation. To illustrate these analytical differences empirically, the paper furthermore applies both observational perspectives to a comparative case illustration of migration and integration administration in Germany, Sweden and France (4.). This comparative re-observation demonstrates more concretely how administrative constellations change in analytical meaning depending on the guiding distinction through which they are observed.

The following reconstruction argues that the ACI framework relies on the distinction of *coordination success* and *coordination failure*. Through this distinction, administrative action appears as the alignment of interdependent decisions and, further, IARs as the structures organizing this alignment. At the same time, this distinction structures the observation of administrative processes by focusing on constellations of interdependent actors whose interactions are institutionally shaped and strategically oriented toward the production of policy outcomes. Within this perspective, IARs become observable as stabilized relations that structure these constellations across organizational and territorial boundaries, including vertical and horizontal interdependencies as well as their institutional embedding and practical handling at administrative interfaces. The employed normative distinction, however, also enables a diagnostic observation of IARs, in which both too dense and insufficient relations are interpreted as sources of coordination failure. Configurations in which additional actors and relations are involved beyond what is considered necessary appear as generating excessive coordination demands, while constellations in which relevant actors are not adequately connected are observed as leading to fragmented processing and unclear responsibilities. IARs thereby appear not only as structural features of multi-level administrative systems, but as indicators for evaluating the adequacy of coordination in policy implementation. In this way, the observation shifts from a descriptive account of interdependencies to a normatively guided assessment of how administrative relations contribute to or hinder the coordination of interdependent decisions.

Building on the IARs made visible through this distinction, SST is mobilized as a further analytical perspective for the observation of the same object, drawing in particular on Luhmann’s (2021b) observation of administration. By productively applying the *system/environment distinction* (Luhmann, 2002b, 83–84), the paper reconstructs public administration as a decision system and shifts the focus from the coordination of actors to the recursive connectivity of decisions. Administrative action becomes observable as the ongoing reproduction of decision-making within environmental complexity, in which organizations must process heterogeneous societal references through internally differentiated decision-making programs. Against this background, IARs are observed as forms through which such decision contexts are related and stabilized. This observation is further grounded in the assumption of a functionally differentiated and polycontextural society, in which multiple, non-integrable environmental references must be processed simultaneously. Within this perspective, IARs are no longer normatively interpreted as coordination solutions or problems but as conditions of organizational decision-making and thereby enable the continuation of administrative action and policy implementation.

This contrast can be related to a more general methodological distinction in the study of organizations. As Vogd (2009a, 7–8) argues, organizational research may either proceed from predefined models of organizational purpose and evaluate performance in terms of given goals, or adopt a reconstructive perspective that refrains from such assumptions and instead seeks to understand how organizations generate and stabilize their own conditions of operation. While not identical, the two perspectives discussed in this paper can be situated along this differentiation: the ACI framework foregrounds the evaluation of coordination in relation to policy outcomes, whereas the SST perspective adopts a reconstructive approach that focuses on the self-referential reproduction of organizational decision-making. By systematically varying the guiding distinctions through which IARs are observed, this paper demonstrates how second-order observation can be used as a reflexive research strategy for reconstructing, comparing and transforming established analytical frameworks through the explicit observation of their guiding distinctions. In this sense, SST is treated not only as a substantively different perspective on administration, but as an observational methodology for reflexively examining how research objects are constituted within certain strands of administrative research. At the same time, this methodological perspective remains grounded in SST’s own theoretical commitments concerning organizations, society and their differentiation.

## 2 Making IARs observable through ACI

ACI is approached here as an observational framework through which IARs become visible (Scharpf, 1997, 30). The following section first provides a brief overview of the employed ACI perspective, then reconstructs how IARs are observed within this framework, before finally explicating the distinction underlying this mode of observation.

The designation of ACI as a framework is a deliberate choice: Scharpf (1997) emphasizes that “it is important to keep in mind that a framework is not a theory” (1997, 37). ACI conceptualizes policy as intentional action by actors. Actors are seen as having “interests and preferences, but their knowledge is bounded and their actions are shaped by culturally and institutionally defined roles, norms and belief systems” (Scharpf, 1997, 21). On this basis, ACI introduces a set of analytical categories through which administrative processes are observed. *Actors* are conceptualized primarily as collective or corporate entities in whose name individuals act. Further, political processes are understood as *actor constellations*, that is, strategic interdependencies among multiple actors. Outcomes emerge not from a single decision center but from negotiation, coordination, or conflict among mutually dependent actors. Third, *modes of interaction* refer to the institutionalized procedures through which decisions are reached within given constellations. The analytical focus of this perspective lies on the production conditions of policy output rather than on its substantive or technical adequacy (Scharpf, 1997, 43). Within this framework, public administration appears as a central empirical domain of analysis (Schreurs, 2023), in which administrative organizations are observed as institutional arrangements that structure the interaction of collective actors and shape the conditions under which policy implementation takes place (Treib, 2023, 253). ACI can be situated within the broader family of neo-institutionalist approaches that combine institutional analysis with action theoretical assumptions (cf. Hall and Taylor, 1996; Immergut, 1998; Schimank, 2007b). At the same time, it represents only one among several approaches to the analysis of public administration. Alongside ACI, perspectives such as New Public Management (Lane, 2000), governance (Benz and Dose, 2004), and multi-level governance (Marks et al., 1996; Hooghe and Marks, 2001) offer alternative ways of conceptualizing administrative processes, each emphasizing different aspects such as efficiency, coordination, or the distribution of authority across levels of government.

The selection of ACI as the object of second-order observation is not intended to privilege it over these alternative approaches. Rather, it follows from the methodological objective of the present paper. The contribution does not seek to compare competing theories of public administration, but to reconstruct the distinctions through which IARs become observable as an analytical object. While different approaches could in principle be subjected to second-order observation, contemporary IAR research is primarily situated within an ACI framework. More specifically, the paper focuses on the IAR framework developed by (Bogumil and Kuhlmann (2022) and Bogumil and Gräfe (2023) because it represents one of the few attempts to conceptualize IARs as a distinct analytical category for studying administrative implementation (cf. Oehlert and Kuhlmann, 2024, 80–81). This makes the ACI-based IAR framework a particularly suitable point of departure for the present second-order reconstruction (cf. Benz, 2025; Kuhlmann et al., 2025).

### 2.1 Observing IARs through the ACI-perspective

Early work in ACI-oriented policy research, most prominently the theory of *Inter-Governmental Relations* developed by Scharpf et al. (1976), Scharpf (1978), and Scharpf (1985), provided a first systematic conceptualization of relations within the politico-administrative system.^2^ The theoretical framework focusses on German federalism and describes it as a system of intertwined decision-making processes between the federal government, the *Länder* and municipalities, characterized by an institutional fragmentation of competences. This perspective established an analytical approach for examining interdependencies between governmental levels but focuses primarily on legislation and political will formation, thereby leaving policy implementation – that is, the process within public administration where interdependence must be practically coordinated – systematically under-observed.

Ellwein (1994, 75) subsequently coined the term *inter-administrative relations*. In *The Dilemma of Administration*, he analyzes the relations between different levels of the German administrative system and draws attention to the relations between them and their implications for practice. His work was taken up by Benz (1997, 2000) who further distinguishes territorial, functional and organizational dimensions of relations within his conceptualization.^3^ While both approaches render interdependencies within the politico-administrative system analytically visible, they do not systematically conceptualize these relations in terms of the coordination of administrative action. A shift occurs with the work of Bogumil and Kuhlmann (2022) and Bogumil and Gräfe (2023), who explicitly develop IARs into an analytical concept centered on policy implementation (cf. Gräfe et al., 2025).

Following these authors, IAR is conceptualized as an analytical approach to administrative action and described as a “missing link” in federalism research (Bogumil and Kuhlmann, 2022). The concept enables the observation of administrative structures and policy implementation by focusing on the interdependencies between administrative actors (Bogumil and Gräfe, 2023). Within this framework, IARs are defined as permanent interorganizational relationships, that is, as stabilized forms of interdependence (Bogumil and Gräfe, 2023, 421). Policy implementation is described as formally regulated interactions and processes of mutual coordination, in which administrative actors make decisions to which others subsequently adjust their own (Bogumil and Gräfe, 2023, 422). Implementation, therefore, becomes observable as an ongoing process of coordinating interdependent decisions, in which autonomy must be balanced with the need for standardization and stability.

Drawing on Benz and Dose (2004), the authors distinguish between *vertical IARs*, which describe dependencies between federal, state and local levels and *horizontal IARs*, which encompass interdependencies between organizations at the same hierarchical level, such as ministries, agencies or municipalities. They further operationalize three dimensions through which IAR is specified as an observational framework: *density of relations*, as a measure of the scope and number of levels and actors involved in implementation, *organizational relations*, as the institutional framing and stabilization of these interdependencies, and *interface management*, as the operative practice of coordination at the relations between administrative bodies, in which IARs prove themselves in the everyday practice of administrative implementation or appear as problematic (Bogumil and Gräfe, 2023, 422–425). Together, these dimensions render empirically observable, where coordination takes place within the administrative system. This emphasis on coordination determines how IARs become observable within the ACI perspective.

The reconstruction so far has outlined how IARs become observable within the ACI perspective through specific analytical categories and dimensions, without yet explicating the distinction that structures the mode of observation. While IARs appear as interdependencies within the politico-administrative system, the framework does not remain limited to a descriptive account of these relations. Rather, the observation of IARs is systematically oriented by an underlying normative distinction that guides how they are interpreted. The following section therefore shifts the focus to the explication of this normative distinction, showing how IARs are observed within the ACI framework as either enabling coordination or producing coordination failure, thereby acquiring a diagnostic and normative function for the analysis of administrative action.

### 2.2 Coordination-distinction in the observation of IARs

While IARs are conceptualized as a structural feature of multi-level administrative systems, they acquire a diagnostic function within the ACI framework by being observed through the distinction of coordination success or coordination failure: Bogumil and Kuhlmann (2022) explicitly differentiate between advantages of IARs (cooperation and coordination) and disadvantages of IARs (lack of transparency, duplication of effort, and a lack of accountability for the overall process), and thereby frame them predominantly in terms of their problematic implications for administrative action (cf. Bogumil and Kuhlmann, 2022, 2). Within this perspective, IAR is treated as a diagnostic tool for evaluating the consequences of administrative relations for policy implementation. *Dense* as well as *insufficient relations* are observed as sources of coordination failure. The observation therefore shifts from administrative structures to a normative observation of *over-* or *under-entanglement* (Bogumil and Gräfe, 2023, 425). Over-entanglement refers to constellations in which additional administrative bodies are involved beyond what is considered necessary for task execution (Bogumil and Gräfe, 2023, 426). This results in an increase of relations that must be processed, without contributing to the functional requirements of implementation. Such configurations are typically attributed to historically evolved allocations of competencies across levels of government or to the extension of organizational jurisdictions in response to resource dependencies and strategic interests. The resulting relations are observed as generating avoidable coordination demands, which burden administrative processes. Under-entanglement, in turn, captures situations in which relevant administrative actors are not incorporated into the relations despite their substantive involvement in the issue at hand (Bogumil and Gräfe, 2023, 427). This exclusion is interpreted as resulting from institutional constraints such as legal barriers to cooperation, rigid organizational demarcations, or actor-specific strategies of non-involvement. In these cases, the absence of necessary relations is observed as leading to fragmented processing, in which interdependencies appear as insufficiently addressed and responsibilities as not clearly assumed.

Bogumil and Gräfe (2023) apply their ACI-grounded observational framework of IARs to various policy fields within the German federal system, employing their normative distinction. Migration and integration policy, social policy and digitalizing public services in Germany are observed as cases in which IARs become observable as problems for coordination of policy implementation. In the field of *migration and integration policy*, implementation structures are described as characterized by fragmented and overlapping competences across federal, state and local authorities. IARs take the form of coordination requirements, particularly in sequential decision-making processes involving multiple actors. Insufficiently coordinated interfaces are reconstructed as coordination failures, manifesting in delays, inconsistent decisions and problems in information exchange between authorities. The density of IARs thereby appears as a source of increased coordination demands and corresponding deficits. In *social policy*, responsibilities are analyzed as fragmented across job centers, social welfare offices and other agencies operating under different legal frameworks. These arrangements generate interdependencies, particularly in cases involving frequent shifts in responsibility and sequential processing across administrative units. Insufficiently coordinated interfaces are observed as coordination failures, described as gaps in benefits during transitions, incomplete case handovers and information losses between organizations, often accompanied by delays and unclear responsibilities. In the context of the *digitalization of public services*, interdependencies emerge from shared digital infrastructures, such as centralized service portals, common registers and jointly developed IT systems across administrative levels. The lack of binding coordination in the development and reuse of these systems is likewise identified as coordination failure, described as parallel development of similar services, limited interoperability between systems and delays in implementation (Bogumil and Gräfe, 2023, 429–440). Across these fields, IARs are systematically observed through the normative distinction between coordination success and coordination failure and thereby appear as either successful or deficient forms of coordinating interdependent administrative actors, with a predominant emphasis on coordination failure.

This analysis shows that IARs are constituted through the guiding distinction of the ACI framework, which becomes visible from the second-order perspective applied here. In this respect, the ACI perspective is analytically productive insofar as it renders IARs observable in the first place by reconstructing them as interdependencies and as either enabling or hindering the coordination of strategically oriented actors across organizational interfaces. At the same time, however, this mode of observation remains bound to their employed normative distinction and thereby frames the phenomenon solely within a problem- or solution-oriented framing. The observation applied here therefore yields more than an account of IARs within the ACI framework. It exemplifies how second-order observation can render the constitutive distinctions of established research approaches explicit and thereby open them to comparison and reflexive transformation.

At the same time, this coordination-oriented diagnosis presupposes a specific understanding of organizations, namely that IARs are to be assessed primarily in terms of their contribution to externally attributed goals of coherent and effective policy implementation. At this point, a more general organization-theoretical reservation becomes relevant. As Vogd (2009a, 8) argues, empirical research cannot assume in advance that it already knows the purpose of an organization; rather, it must remain open to the possibility that organizational performance may consist precisely in evading excessive goal expectations. This shifts attention from goal attainment to the ways in which organizations stabilize themselves under conditions of potentially overburdening demands. IARs, accordingly, do not necessarily appear as deficits or achievements of coordination, but can also be understood as organizational arrangements that contribute to maintaining decision-making capacity. The following section builds on this by observing IARs within an SST framework centered on the self-referential reproduction of administrative organizations (Nassehi, 2005). Within this perspective, IARs are no longer observed in terms of coordination success or failure, but through the distinction between system and environment, which makes observable how administrative organizations reproduce themselves through their own decision-making operations under conditions of environmental complexity.

## 3 Observing IARs through SST

Building on the preceding second-order observation, this chapter reconstructs IARs through a different guiding distinction, thereby reconstituting the object in analytical terms. This reconstruction is grounded in SST’s own theoretical assumptions concerning the self-referential reproduction of organizations, the functional differentiation of society and the system/environment distinction. Accordingly, the following analysis does not proceed from a neutral methodological standpoint but reconstructs IARs through a specific systems-theoretical perspective whose own distinctions and assumptions remain contingent and therefore open to reflexive observation.

Observing IARs through SST presupposes a fundamental shift in what is seen as the basic operation of administration. Luhmann (2021b, 74) develops his theory of public administration as a theory of decision systems. Rather than focusing on actor coordination, SST observes decisions as the elementary operations through which organizations act (Luhmann, 2002a, 398; Luhmann, 2021b, 31). Administrative action becomes observable as the recursive production of decisions within organizations that process multiple environmental references. Decisions function as the central operations through which organizational structures are enacted, while expectations define the horizon of possible connections between decisions (Luhmann, 2021b, 71).

By employing a system/environment distinction, SST enables a reconstruction of IARs as interdependencies between organizational decision contexts. This shift is analytically productive insofar as it makes observable aspects of IARs that remain within the blind spots of a coordination-oriented perspective, namely the organizational function of such relations. The system/environment distinction directs attention to the conditions under which administrative decision-making is possible – the function “for the constancy or the continuity of organizations” (Nassehi, 2005, 179) – allowing IARs to be observed as forms that stabilize connectivity across heterogeneous decision contexts. At the same time, by refraining from *a priori* normative assumptions, this perspective enables a more differentiated analysis of IARs, in which relations are not immediately interpreted in terms of deficits or improvements, but can be reconstructed with regard to their contribution to the production of organizational decision-making. The perspective is therefore not limited to the present case. It indicates how systems-theoretical observation can contribute to reflexive empirical research by reformulating established objects of inquiry in terms of alternative distinctions and by making the contingency of prior problem framings visible.

An SST perspective on administration thereby focuses on the internal selection logics and mechanisms of organizational (re)production by employing a system/environment distinction, within which IARs emerge as connecting decision contexts. This perspective is analytically unfolded in two steps: first by reconstructing the internal differentiation of administrative decision contexts and subsequently by showing how these differentiated contexts become related through the processing of multiple environmental references, thereby constituting IARs.

### 3.1 Organizational differentiation as a precondition of IARs

By employing the system/environment distinction as an observational form, organized systems can be reconstructed as constituting themselves in difference to an environment that is accessible only in relation to them. The environment is therefore not treated as an objectively given external reality that can simply be represented within the organization. Rather, it becomes relevant only insofar as it is selectively processed through the system’s own operations. From this perspective, organizations do not confront a single, unified environment. The environment instead appears as a multiplicity of heterogeneous and potentially conflicting references that cannot be integrated into one coherent decision logic. Different societal expectations, institutional demands and problem definitions simultaneously address the organization without converging into a single rationality. These references become relevant for the organization only insofar as they are internally processed in the form of irritations (Luhmann, 2019, 346). The analytical focus thereby shifts from the environment itself to the organizational processing of environmental complexity. Systems-theoretical observation does not ask how organizations accurately represent their environment, but how they maintain their own capacity for decision-making.

This condition can be described in systems-theoretical terms as the necessity to generate internal complexity in order to process environmental complexity (Luhmann, 2022, 318; Luhmann, 2024, 121). Internal differentiation thereby becomes a precondition for maintaining organizational decision-making capacity under conditions of (increasing) complexity.^4^ What appears as internal complexity is thus not a one-to-one representation of an objectively given environment, but can be reconstructed as the result of the system’s own productive differentiation from an operatively inaccessible environment (Luhmann, 2000, 314). From an empirical perspective, this differentiation is, however, not simply a structural response to environmental complexity, but also a way of limiting the mutual irritation of incompatible expectations. Organizations develop stabilizing practices that prevent an overload of decision-making by structuring which references become relevant in specific contexts and which are temporarily ignored (Vogd, 2009a, 13).

Administration is seen as processing societal differentiation within its own decision-making operations, insofar as the environment is only accessible within the system through distinctions drawn by the system itself (Luhmann, 1989). From the applied perspective, administrative organizations rely on internal differentiation in order to stabilize decision-making capacity. This becomes visible in the policy fields discussed by Bogumil and Kuhlmann (2022) and Bogumil and Gräfe (2023), where migration, social policy and digital administration illustrate how heterogeneous societal references are translated into organization-specific decision programs. Accordingly, administrative organizations can be reconstructed as developing differentiated decision programs that coexist within the same organization. Such internal differentiation can be interpreted as a way of stabilizing decision-making capacity by maintaining the system/environment distinction within organizational operations. Administration thus appears as sustaining its capacity to decide by further elaborating its own decision programs, resulting in structures that can be described as multiple decision programs operating in parallel.

This mode of observation, therefore, reconstructs administrative organizations as internally differentiated systems. However, it does not yet specify how these differentiated decision contexts become related to one another, namely what constitutes IARs. Within the ACI framework this is explained as the outcome of coordination processes between strategically interdependent actors. The following section reconstructs the conditions under which such relations emerge by showing how the necessity to relate differentiated decision contexts arises from the processing of multiple environmental references.

### 3.2 IARs and multiple environmental references

The ACI perspective reconstructs administrative action as dependent on interdependent actors whose actions must be coordinated. What remains unobserved in this framing is that these interdependencies can be traced back to a societal environment that confronts administrative organizations with heterogeneous references (Schimank, 2005, 43). Organizations take up irritations from different societal function systems and process them according to their own decision premises (Luhmann, 2019, 386). In this way, distinct organization-specific forms of issue processing emerge, which operate according to different logics without the presence of a dominant coordinating instance.

Modern society is observed within SST as functionally differentiated. Society does not operate according to a single hierarchical order or a unified societal rationality. Instead, different societal domains process communication according to their own specific distinctions and operational logics. Against this background, the same societal issues can simultaneously become relevant within multiple communicative contexts without being reducible to one overarching perspective. Different societal domains process these issues according to different distinctions and criteria of relevance (Baecker, 2017, 110). Within SST this is described as polycontexturality (Luhmann, 2021a, 892). The concept points to the coexistence of multiple contextures, that is, multiple simultaneously operative horizons of observation and meaning-processing. Society operates through a plurality of distinctions rather than through one universally shared perspective (Luhmann, 2002b, 666). Different communicative contexts process events according to different criteria of relevance. As a result, the same events can simultaneously generate multiple and potentially conflicting references (Schimank, 2005, 54–55).

However, this perspective does not yet specify implications at the level of organizations. Its relevance for public administration emerges where the organization processes this societal polycontexturality operatively. Scharpf (1971, 21) rightly emphasizes that societal problem contexts do not adhere to jurisdictional boundaries within public organization, but rather that, as scientific, technological and economic developments accelerate, the range of demands directed at the politico-administrative system grows, resulting in a rising volume of issues to be handled. At the same time, these issues become increasingly interrelated, forming more extensive and tightly interconnected constellations of problems that extend across previously distinct domains. As a result, organizations are confronted with demands that cannot be reduced to a single, unified logic.^5^ For administrative organizations, this means that internally differentiated decision-making programs cannot remain isolated. The coexistence of differentiated decision contexts is therefore supplemented by a requirement to establish connections between them in order to maintain the continuity of decision-making. It is under these conditions that relations between differentiated decision programs emerge as a necessary organizational response.^6^ IARs are thus observed as forms through which differentiated decision programs are related in the course of processing heterogeneous environmental references. They arise from the necessity to connect otherwise separate decision contexts under conditions of polycontexturality. In this perspective, IARs do not appear as coordination problems, but as conditions for the continuation of organizational decision-making within a functionally differentiated society.

What appears within the ACI framework as excessive or insufficient relations is therefore not a property of administrative action, but the effect of an observation guided by the coordination-distinction. Observed through the system/environment distinction, the same relations appear as organizational forms that emerge from the requirement to process multiple environmental references and to maintain connectivity across differentiated decision contexts. These observations can be related back to the empirical examples, namely migration and integration policy, social policy and the digitalization of public services. In these fields, the coordination problems identified within the ACI framework – such as fragmented responsibilities and sequential decision dependencies in migration administration, discontinuities and shifting responsibilities in social policy, or interoperability deficits and parallel system development in digital administration – can from this perspective be observed as effects of the necessity to process multiple environmental references.^7^ What appears as insufficient or excessive coordination thus becomes observable as the outcome of relating differentiated decision contexts that are structured by heterogeneous societal demands (for a comparative visualization see Table 1).

**Table 1:** Comparative overview of ACI and SST in the observation of IARs.

Comparative dimension	ACI	SST
Guiding distinction	Coordination success/coordination failure	System/environment
Analytical focus	Coordination of interdependent actors	Reproduction of organizations through decisions
IARs	Problems or solutions of coordination	Conditions of stabilizing connectivity across decision contexts

While the ACI framework can reconstruct coordination deficits, fragmented implementation structures and institutional interdependencies with considerable analytical precision, persistent overlaps, delays and fragmented responsibilities can only appear within its coordination-oriented distinction as deficiencies of implementation. Since the analytical focus remains tied to the question of whether coordination succeeds or fails, the possibility that these same arrangements may simultaneously contribute to stabilizing organizational decision-making across differentiated administrative contexts largely escapes the observational frame itself. The SST perspective, by contrast, makes visible how such relations may function to maintain connectivity between differentiated decision contexts under conditions of heterogeneous societal demands and functional differentiation. Building on this comparison, the following section provides a more systematic comparative application of both observational perspectives by re-observing an empirical example reconstructed within the ACI framework. Through this focused illustration, the analytical shift from coordination-oriented observation to the observation of differentiated decision-making contexts can be demonstrated more concretely.

## 4 Comparative re-observation of IARs in migration and integration administration

The analytical implications of the different observational distinctions developed in the preceding chapters become particularly visible when applied to a concrete empirical field. The following section therefore provides a focused comparative case illustration of how different observational distinctions reconstruct the same administrative constellations differently. Drawing on a comparative analysis of migration and integration administration in Germany, Sweden and France developed by Oehlert and Kuhlmann (2024) the section demonstrates how IARs become observable within the coordination-oriented distinction between coordination success and coordination failure.^8^ Comparable dynamics have also been described within broader international debates on migration governance and interorganizational administration (cf. Scholten and Penninx, 2016; Bogumil et al., 2023). The section subsequently shows how the analytical meaning of these same administrative relations changes when re-observed through the system/environment distinction.

In Germany, asylum procedures conducted by the Federal Office for Migration and Refugees (BAMF), subsequent residence-related decisions by local immigration offices, welfare provision by municipal social administrations and access to labor-market integration and integration services are linked through sequentially dependent administrative procedures (Bogumil and Gräfe, 2023, 429–433; Oehlert and Kuhlmann, 2024, 87). Municipal administrations must react to refugee allocations by the *Länder*, organize accommodation, registration, healthcare provision and welfare support, while immigration offices depend on prior BAMF asylum decisions for issuing residence permits. Delays in asylum procedures therefore affect subsequent residence-related processing, welfare provision, labor-market access and participation in integration programs. In Sweden, municipalities, the Swedish Migration Agency, employment services and county administrative boards are interconnected through extensive coordination requirements concerning refugee distribution, housing allocation, labor-market integration and information exchange (Oehlert and Kuhlmann, 2024, 88–89). Municipalities are required to organize accommodation for recognized refugees within short timeframes while simultaneously depending on central agencies for allocation decisions and timely information concerning refugee arrivals. Local representatives describe existing coordination mechanisms as partially deficient due to delayed or insufficient information exchange and unclear responsibilities regarding integration-related tasks (Oehlert and Kuhlmann, 2024, 89). In France, integration policy is structured through dense vertical relations between central state actors, prefectures and local authorities, particularly within contractualized coordination arrangements such as the CTAIR (Contrat Territorial d’Acceuil et d’Intégration des Réfugiés) system (Oehlert and Kuhlmann, 2024, 90–91). Although integration-related responsibilities remain strongly centralized through actors such as the French Immigration and Integration Office (OFII) and the prefectures, local administrations increasingly assume complementary and compensatory functions where state-led integration structures prove insufficient, particularly regarding vulnerable migrant groups, local welfare provision and territorially specific integration support.

Observed through the SST perspective, however, the analytical meaning of these same administrative constellations changes. The BAMF does not process *integration* as a unified societal objective, but asylum-related legal recognizability. Immigration offices process residence-related legality and documentation, municipal welfare administrations process eligibility for social benefits and accommodation, labor market agencies process employability, and municipalities process territorially specific responsibilities concerning housing, schooling, health care provision, and local integration infrastructures. The administrative interfaces described above therefore emerge because decisions produced within one organizational context become necessary preconditions for subsequent decisions in other organizations while simultaneously remaining tied to different organizational programs and decision premises. Fragmented responsibilities and intensified coordination requirements consequently no longer appear primarily as failures of coordination between administrative actors. Rather, they become observable as effects of relating differentiated decision-making contexts that cannot be integrated into a single unified administrative logic under conditions of heterogeneous societal demands.

From this perspective fragmented responsibilities and intensified coordination requirements do not merely indicate insufficient coordination between administrative actors. Rather, they reflect the ongoing organizational necessity to stabilize decision-making across differentiated administrative contexts operating according to partially incompatible decision premises. IARs thereby appear as mechanisms through which differentiated organizations maintain operational continuity while simultaneously processing multiple societal demands across administrative levels and policy fields. The comparative cases demonstrate that the persistence and intensification of IARs across different national administrative traditions cannot be sufficiently explained in terms of coordination success or failure. They also point to the organizational function of such relations in maintaining the continuity of administrative decision-making under conditions of functional differentiation and societal complexity. In this respect, the reconstruction also establishes connections to broader international debates on institutional complexity, interorganizational governance and policy networks, in which dense coordination structures and persistent interdependencies are increasingly reconstructed not merely as implementation deficits, but as structural conditions through which governance arrangements remain operable under complex societal demands (cf. Provan and Kenis, 2008).

## 5 From coordination problems to complexity processing

The preceding chapters reconstructed inter-administrative relations as an object constituted through distinct observational frameworks by applying a second-order observational methodology. This approach makes the guiding distinctions of administrative studies within this research tradition analytically accessible and open to variation. By systematically varying these distinctions, the paper has shown that IARs are not merely described differently but transformed in their analytical meaning as a function of the distinctions through which they are observed.

Within this methodological framework, the ACI-based perspective on IARs developed by Bogumil, Kuhlmann and Gräfe relies on the distinction between coordination success and coordination failure. The ACI approach provides the categories through which IARs become empirically observable. By focusing on actor constellations, institutional constraints and modes of interaction within policy implementation, it reconstructs IARs as interdependencies that organize administrative action. Within this frame, IARs appear as arrangements whose density, organizational relations and interface management can be assessed in terms of their contribution to successful coordination. This observation extends into the reform proposals developed by the authors (Bogumil and Kuhlmann, 2022, 5; Bogumil and Gräfe, 2023, 428). Strategies such as harmonization, hierarchical steering and the reconfiguration of responsibilities are formulated in response to identified coordination requirements, while their realization is constrained by existing interdependencies, political conditions and organizational self-interests. The distinction guiding the observation of IARs therefore persists by structuring both the identification of coordination potentials and the diagnosis of coordination failures. In this way, the ACI perspective provides a differentiated empirical account of administrative interdependence. At the same time, however, it remains limited to the normative coordination-based distinction through which it renders IARs visible. It can account for how coordination succeeds or fails within policy implementation, but not why such relations persist despite recurring analysis of coordination problems or what function they fulfil for maintaining organizational decision-making. This became particularly visible in the comparative reconstruction of asylum procedures, welfare provision and refugee allocation in Germany, Sweden and France, where dense interdependencies and recurring coordination requirements persisted despite being repeatedly described as problematic within administrative practice.

This limitation becomes particularly visible when considering empirical observations of organizational practice that cannot be adequately captured within a coordination-oriented framework. Further neo-institutionalist research has pointed to mechanisms through which organizations stabilize their operations under conditions of complex and often overburdening expectations. These mechanisms have been described in terms of decoupling, that is, the selective separation of formal structures and representations from actual performance processes (cf. Powell and DiMaggio, 2012). As Vogd (2009a) notes, this may include arrangements in which evaluation primarily addresses documentation rather than underlying practice. What appears from a coordination-oriented perspective as insufficient control or ineffective coordination can thus be reconstructed as a way of limiting mutual irritation between heterogeneous expectations. The comparative cases illustrated this particularly clearly in the sequential dependencies between asylum decisions, municipal welfare provision and residence-related processing, where delays, fragmented responsibilities and intensified coordination requirements emerged from the necessity to relate differentiated organizational decision premises across multiple administrative contexts. In this sense, such configurations do not necessarily indicate a lack of coordination but can be understood as part of the organizational conditions that enable the continuation of decision-making.

Against this background, the paper reconstructed the same relations through a different guiding distinction grounded in SST. More specifically, it draws on Luhmann’s (2021b) analysis of Administration, which employs the distinction between system and environment. Through this shift, IARs become observable as organizational forms that stabilize decision-making and enable the processing of multiple, non-integrable environmental references under conditions of polycontexturality, that is, the societal structure in which heterogeneous references cannot be integrated into a single decision logic. From this perspective, the reconstruction does not evaluate IARs in normative terms; instead, they become observable as structural conditions of administrative decision-making within modern society. They function as mechanisms that stabilize connectivity across differentiated decision contexts and thereby contribute to the recursive self-reproduction of the administrative system. This analytical shift makes visible the organizational function of IARs. It is not merely a variation in perspective but entails a change in the elementary operation through which administrative processes are observed, moving from the coordination of actors to the recursive connectivity of decisions and thereby redefining the unit of analysis.

However, following the logic of second-order observation itself, the SST reconstruction also remains bound to its own guiding distinction and therefore produces corresponding blind spots. The systems-theoretical reconstruction therefore does not provide a privileged or theoretically neutral perspective on IARs, but remains tied to the specific assumptions of SST concerning organizations, society and decision-making. By focusing on decision-making as the elementary operation of organizations and on the processing of environmental complexity, SST renders visible dimensions of administrative reproduction that remain less accessible within ACI. At the same time, the analytical focus shifts away from dimensions that are central to the ACI perspective. Questions concerning actor constellations, strategic interaction, intentional coordination and variations in implementation outcomes become less visible. Consequently, SST is less suited to explaining why some administrative interfaces are associated with delays, information losses and unclear responsibilities while others are not, or how specific actors and coordination practices contribute to these differences. Likewise, SST does not provide criteria for evaluating IARs in normative terms, for example with regard to coordination success, implementation quality or administrative performance. These dimensions remain more directly observable within the ACI framework, which is specifically designed to analyze actor constellations, coordination processes and implementation outcomes. The two perspectives therefore do not simply provide competing explanations of the same object, but render different aspects of IARs visible while simultaneously generating different blind spots. The relationship between the two perspectives is therefore neither one of simple substitution nor one of cumulative complementarity. Because they rely on different guiding distinctions, ACI and SST constitute IARs in analytically different ways and therefore cannot be reduced to one another. At the same time, they remain comparable insofar as they are applied to the same administrative constellations and render different dimensions of these constellations observable. The contribution of second-order observation lies precisely in making these alternative constructions of IARs reflexively accessible and comparable.

The contribution of this paper thus lies in relating these two frameworks in a way that makes observable how IARs change their analytical meaning from coordination problems to conditions of organizational decision-making. Through this double observation, the same constellation appears both as a coordination problem or solution and as a condition for the continuity of organizational decision-making. The ACI perspective provides the analytical means to empirically specify IARs as interdependencies and to diagnose coordination problems within policy implementation. The SST perspective, by contrast, observes these relations in terms of their function for the reproduction of organizations under conditions of environmental complexity. It thereby becomes possible to explain why IARs persist as organizationally stabilizing forms.

By reconstructing IARs through the system/environment distinction, this paper also connects to broader international discussions on organizations, interdependence and governance under conditions of complexity (Scott, 2004). Since the emergence of open-system perspectives, organizational analysis has increasingly moved beyond closed, entity-based models and has emphasized how organizations are shaped by complex environments, interdependence and ongoing processes of organizing (Thompson, 1967; Lawrence and Lorsch, 1973). Research on institutional environments, organizational fields and network forms has further shown that organizations are embedded in heterogeneous expectation structures (Meyer and Rowan, 1977) and increasingly depend on relations among autonomous but interdependent organizations rather than on hierarchical steering alone (Powell, 1990; Provan and Kenis, 2008). The reconstruction developed in this paper contributes to these discussions by re-observing administrative interdependencies as forms through which differentiated organizational decision contexts maintain connectivity under heterogeneous societal demands. As the illustrative empirical analysis demonstrated, dense administrative interdependencies and recurring coordination pressures may persist not merely as indicators of coordination success or failure, but also as organizational conditions for maintaining decision-making across differentiated administrative contexts. Reform proposals and diagnoses of coordination problems can therefore be understood as effects of specific observational distinctions rather than as direct responses to objectively given deficiencies. Observing how different distinctions constitute IARs in divergent ways makes it possible to relate empirical diagnoses and reform strategies to their underlying analytical premises and to examine how alternative distinctions may transform both problem descriptions and proposed solutions.

## Footnotes

1 Although the observation applied here could, therefore, in principle be described as observing a second-order observer, this paper deliberately refrains from extending the hierarchical language of observation orders any further, since the analytical point lies less in assigning ever higher orders than in making the guiding distinctions of observation explicit. The framework is treated here as an observational instrument whose guiding distinction determines what becomes visible (Vogd, 2009b, 100).

2 Although the ACI framework was only explicitly formulated later, Scharpf (1997, 1) states that it had already been implicitly used in his and Mayntz’s work since the early 1970s.

3 Benz’s (2021) later comparative work further expanded this perspective beyond the German federal context, including analyses of multi-level administrative arrangements in Germany, Australia and Switzerland.

4 Luhmann (2005, 60) describes the environmental complexity that necessitates internal differentiation as historically increasing, a development that becomes visible in a *growing politicization* and *interdependence of interests* (cf. Luhmann, 2000, 70).

5 Bogumil and Gräfe (2023, 421) explicitly draw on Uwe Schimank’s concept of mutual adjustment to characterize implementation processes in the field of integration administration. Coordination problems arise precisely from the fact that each participating administrative unit makes its own decisions, to which other actors must adapt in a responsive manner. This observation corresponds to Schimank’s (2007a) diagnosis of a polycontextural differentiation: administrative decisions are not negotiated within a unified framework of system rationality, but unfold across a plurality of context-specific communicative logics that mutually irritate one another.

6 These contexts, differentiated through distinct binary codes, no longer form a neatly separable order, which calls into question whether the concept of functional differentiation alone sufficiently captures this form of societal structuring (cf. Schimank, 2005, 55).

7 Similar dynamics resulting from the processing of heterogeneous environmental references have been described in the context of healthcare administration (cf. Männle and Feißt, 2025).

8 The study is particularly suitable for the present reconstruction because it explicitly applies the established IAR framework discussed above, while simultaneously addressing a field in which research on IARs remains comparatively limited (cf. Oehlert and Kuhlmann, 2024, 78).
